# Response of Total (DNA) and Metabolically Active (RNA) Microbial Communities in *Miscanthus × Giganteus* Cultivated Soil to Different Nitrogen Fertilization Rates

**DOI:** 10.1128/spectrum.02116-21

**Published:** 2022-02-16

**Authors:** Jihoon Yang, Jaejin Lee, Jinlyung Choi, Lanying Ma, Emily A. Heaton, Adina Howe

**Affiliations:** a Department of Agricultural and Biosystems Engineering, Iowa State University, Ames, Iowa, USA; b Center for Advanced Bioenergy and Bioproducts Innovation, Urbana, Illinois, USA; c Department of Agronomy, Iowa State University, Ames, Iowa, USA; University of Massachusetts Amherst

**Keywords:** activity, *Miscanthus × giganteus*, nitrogen, RNA, soil microbiome

## Abstract

*Miscanthus* × *giganteus* is a promising high-yielding perennial plant to meet growing bioenergy demands; however, the degree to which the soil microbiome affects its nitrogen cycling and subsequently, biomass yield remains unclear. In this study, we hypothesize that contributions of metabolically active soil microbial membership may be underestimated with DNA-based approaches. We assessed the response of the soil microbiome to nitrogen availability in terms of both DNA and RNA soil microbial communities from the Long-term Assessment of Miscanthus Productivity and Sustainability (LAMPS) field trial. DNA and RNA were extracted from 271 samples, and 16S small subunit (SSU) rRNA amplicon sequencing was performed to characterize microbial community structure. Significant differences were observed in the resulting soil microbiomes and were best explained by the sequencing library of origin, either DNA or RNA. Similar numbers of membership were detected in DNA and RNA microbial communities, with more than 90% of membership shared. However, the profile of dominant membership within DNA and RNA differed, with varying proportions of *Actinobacteria* and *Proteobacteria* and *Firmicutes* and *Proteobacteria*. Only RNA microbial communities showed seasonal responses to nitrogen fertilization, and these differences were associated with nitrogen-cycling bacteria. The relative abundance of bacteria associated with nitrogen cycling was 7-fold higher in RNA than in DNA, and genes associated with denitrifying bacteria were significantly enriched in RNA, suggesting that these bacteria may be underestimated with DNA-only approaches. Our findings indicate that RNA-based SSU characterization can be a significant and complementing resource for understanding the role of soil microbiomes in bioenergy crop production.

**IMPORTANCE** *Miscanthus* × *giganteus* is a promising candidate for bioeconomy cropping systems; however, it remains unclear how the soil microbiome supplies nitrogen to this low-input crop. DNA-based techniques are used to provide community characterization, but may miss important metabolically active taxa. By analyzing both DNA- and actively transcribed RNA-based microbial communities, we found that nitrogen cycling taxa in the soil microbiome may be underestimated using only DNA-based approaches. Accurately understanding the role of microbes and how they cycle nutrients is important for the development of sustainable bioenergy crops, and RNA-based approaches are recommended as a complement to DNA approaches to better understand the microbial, plant, and management interactions.

## INTRODUCTION

The sterile allopolyploid (2n = 3x = 57) *Miscanthus × giganteus* (Greef et Deu.) is a promising perennial grass bioenergy crop because of its ability to produce large amounts of biomass with little fertilizer compared with hay or grain crops (1–4). The peak biomass production of *M.* × *giganteus* has been observed to be up to three times higher than switchgrass (*Panicum virgatum* L. cv. Cave-in-Rock), similar to willow (*Salix schwerinii* E. Wolf × viminalis L.), three times higher than reed canary grass (*Phalaris arundinacea* L.), and two times higher than triticale (*Triticosecale* Wittmack) (5–7). Additionally, *M.* × *giganteus* production has lower requirements of nitrogen compared with triticale and reed canary grass, and pesticides compared with cockspur grass (*Echinochloa crus-galli* (L.) Beauv) and reed canary grass (7–10), in addition to reduced nitrate leaching relative to other bioenergy crops (11, 12). These advantages of *M.* × *giganteus* and its ability to maintain high productivity for up to 20 years compared with other energy crops have results in its interest as a bioenergy crop (9, 13–16).

To support its growth, environmental and management factors that can affect the productivity of *M.* × *giganteus* have been evaluated. Previously, *M.* × *giganteus* has been observed to decrease in productivity at low temperatures (17, 18). It has also been observed to have relatively high water demand (19, 20) and to require cultivation for at least 3 years to obtain adequate yield (18, 21–27). Recommendations for nitrogen fertilization of *M.* × *giganteus* are inconsistent, with previous studies showing that fertilization can have little to no effect (28–33) or positively contribute to its productivity (34–37).

Previously, it has been estimated that *M.* × *giganteus* can obtain 16% of its nitrogen demand from the atmosphere during the growing season (38). The nitrogen is provided through the activity of nitrogen-fixing bacteria in the rhizobiome of *M.* × *giganteus* (39), which are enriched early after *M.* × *giganteus* planting (38). Nitrogen fixation genes have been observed to be more abundant in *M.* × *giganteus* relative to other energy crops planted in similar soils (40, 41). Specific phyla which have been identified in *M.* × *giganteus* rhizobiomes include *Actinobacteria* and *Proteobacteria*, which include known nitrogen-fixing families such as *Hyphomicrobiaceae*, *Bradyrhizobiaceae*, *Rhodospirillaceae*, and *Geobacteraceae* (42).

To date, all studies of *M.* × *giganteus* soil microbial communities and their response to fertilization or biomass production have been limited to the characterization of soil environmental DNA. We previously used sequencing of 16S rRNA genes in DNA to identify significant interactions between microbial diversity, stand age, fertilization, and above-ground biomass in *M.* × *giganteus* (43). However, it is possible that DNA-based analysis may underestimate the number of active taxa, resulting in biased interpretations of how microbial communities respond to the environment (44, 45). By contrast, RNA-based characterization of microbial communities, representing metabolically active or transcribed genes, can better relate community responses to environmental variability (46–50). Additionally, RNA-based studies are more sensitive and have detected underrepresented active bacteria that are below the amplification threshold of DNA-based approaches. Despite the advantages of RNA-based methods, direct comparison of the DNA and RNA methods for microbial community characterization in bioenergy crops soil microbial communities is sparse. One previous study of the bioenergy grass, *Pennisetum purpure*, compared bacterial communities of DNA- and RNA-based denaturing gradient gel electrophoresis (DGGE) profiles and clone libraries, and found that RNA-based methods could identify enriched metabolically active membership (51).

In this study, we perform comparison of DNA and RNA approaches to help us better understand how soil microbiome in field-grown *M.* × *giganteus* can inform management and environmental impacts of *M.* × *giganteus* production. We evaluate the effects of stand age (representing different initial growth environments) and fertilization (representing different N availability) on changes in microbial community membership and structure. We hypothesize that microbiome responses (as indicated by DNA and RNA) to *M.* × *giganteus* management will differ and, specifically, that metabolically active (RNA) microbial communities will show a more rapid and sensitive response to fertilization than total (DNA) microbial communities. To test these hypotheses, soil samples were collected from the LAMPS site, a replicated chronosequence field previously used to investigate the effects of stand age and nitrogen fertilizer on *M.* × *giganteus* and corn (Zea mays L.) (30, 52). DNA and RNA were extracted from these soil samples, and we compared these microbial responses with stand age, N fertilization amount, and time since fertilization.

## RESULTS

### DNA and RNA microbial communities differ in microbial composition and alpha diversity.

**FIG 1:**
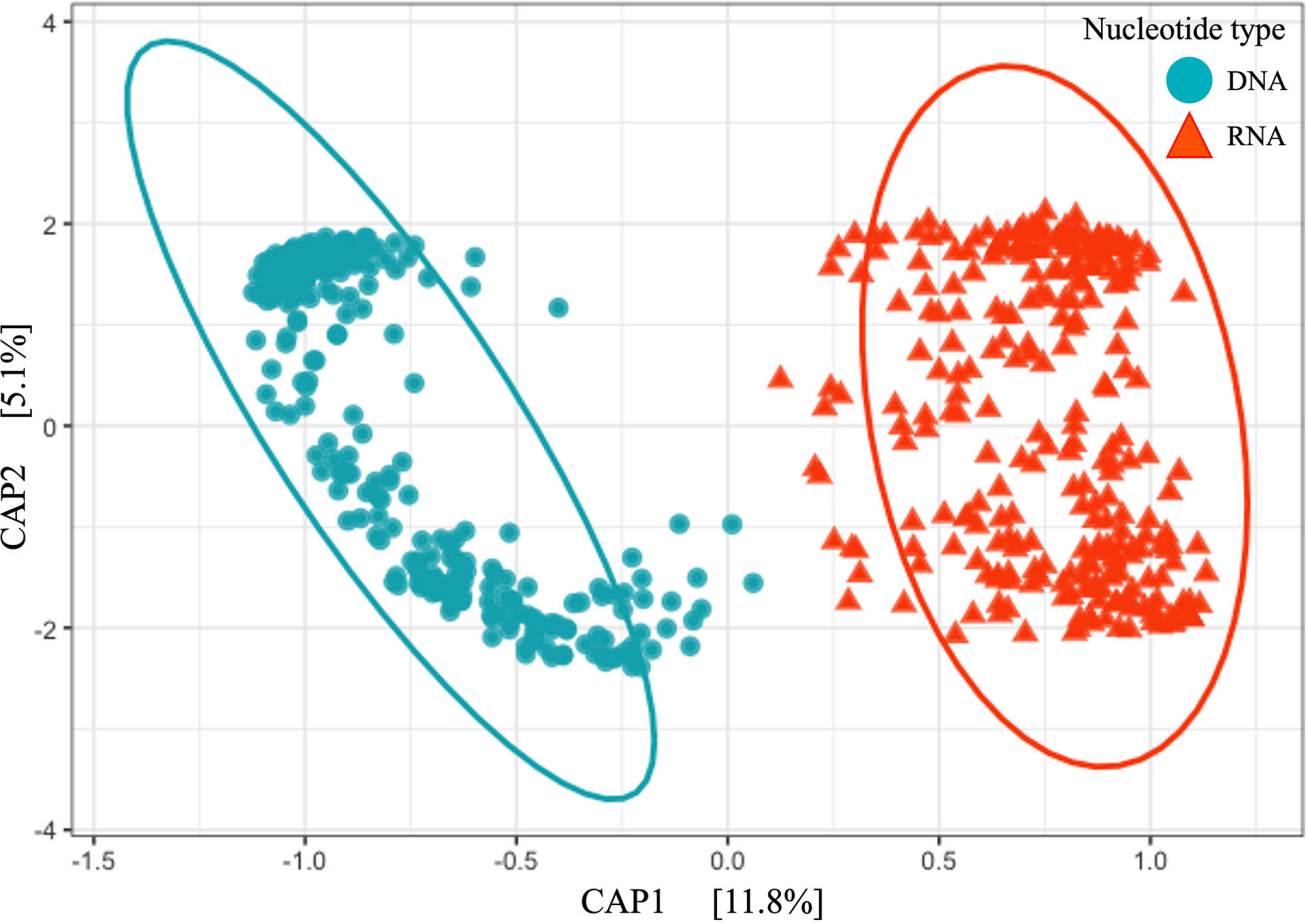
Similarities, assessed with Bray-Curtis indices, between DNA and RNA microbial communities from *M.* × *giganteus* soils. Constrained analysis of principal coordinates (CAP) was used to ordinate Bray-Curtis indices calculated with ASVs. Blue dot and red triangle represent the DNA and RNA microbial communities, respectively.

The DNA and RNA 16S rRNA amplicons from soil samples representing three ages and three fertilization rates of *M* × *giganteus* were compared. The origin of the sequencing library, either DNA or RNA, was found to have the greatest influence on the separation of the microbial community (R^2^_PERMANOVA_ = 0.117, p_PERMANOVA_ = 0.001, Table S1). These differences between DNA and RNA communities were also observed for each sampling day (p_PERMANOVA_ = 0.001). Their influence was 3.9 times higher than that of stand age (R^2^_PERMANOVA_ = 0.030, p_PERMANOVA_ = 0.001) and 15 times higher than that of N fertilization amount (R^2^_PERMANOVA_ = 0.008, p_PERMANOVA_ = 0.001). DNA and RNA microbial communities were observed to separate into clear clusters using constrained analysis of principal coordinates (CAP) along the first axis of the CAP (CAP1, 11.8%, F = 77.56, p_ANOVA_ = 0.001, Fig. 1). The microbial composition (ADONIS, p_ADONIS_ = 0.001) and homogeneity (betadisper, p_betadisper_ = 0.001) of DNA and RNA communities were also observed to be significantly different.

**FIG 2:**
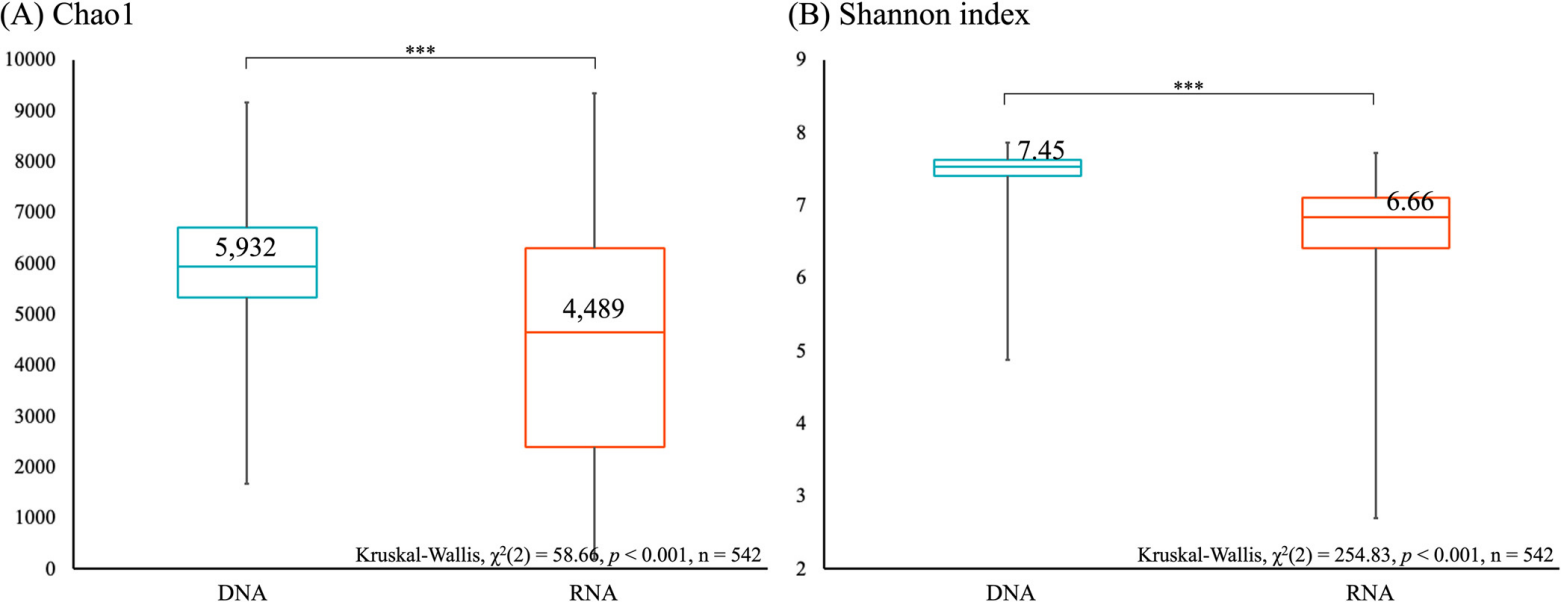
Alpha diversity indices of DNA and RNA microbial communities. Richness, (A) Chao1, (B) Shannon index, were estimated for microbial communities with ASVs. “***” denotes significant differences of alpha diversity indices between DNA and RNA microbial communities at a *P*-value < 0.05 as assess by Kruskal-Wallis with *post hoc* Dunn’s test.

Alpha diversity of soil microbial communities was compared using the Shannon index, which evaluates both microbial richness and evenness, and Chao1, which evaluates the abundance of observed species. Both alpha diversity indices showed significant differences between DNA and RNA microbial communities, with higher alpha diversity observed in DNA microbial communities (p_Shannon_ < 0.001, p_Chao1_ < 0.001, Fig. 2). On average 32% and 12% higher Chao1 and Shannon indices, respectively, were observed in DNA compared with RNA microbial communities. The average value of alpha diversity was higher in DNA, but the variation in alpha diversity indices was larger between RNA samples. Specifically, the DNA Chao1 index was in the range of 1,667 to 9,170, and RNA was associated with a much wider range of 195 to 9,343. Similar results were observed with Shannon indices, with DNA ranging from 4.88 to 7.85 and RNA from 2.69 to 7.72.

### Taxa distributions varied between DNA and RNA microbial communities.

**FIG 3:**
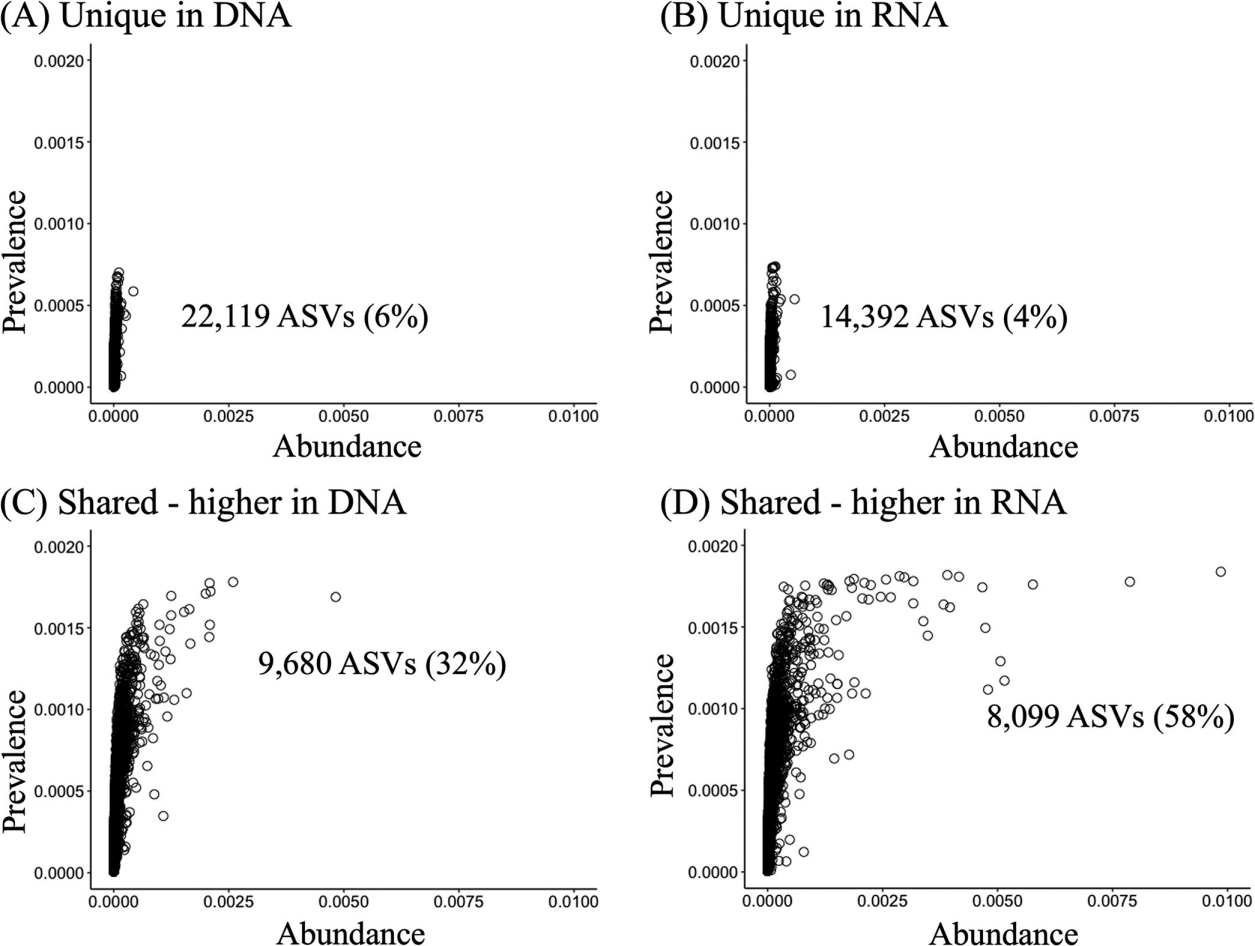
Abundance-occupancy comparison of ASVs in the DNA and RNA microbial communities. Abundance-occupancy distributions were assessed to identify the dynamics of the DNA and RNA microbial community memberships. Each point is an ASV. The ASVs were classified as (A) unique in DNA or (B) unique in RNA, respectively, when it was detected only in the DNA or RNA microbial communities. ASVs detected in both DNA and RNA microbial communities were classified as “shared” and further classified by the average relative abundance based on its enrichment in (C) DNA or (D) RNA.

The total number of taxa in DNA and RNA microbial communities was estimated by observations of amplicon sequence variants (ASVs), where a total of 39,898 and 32,171 ASVs were identified in DNA and RNA, respectively. We compared the ASVs between DNA and RNA samples and found that 17,779 ASVs were shared between DNA and RNA microbial communities (32% and 58%, respectively); 22,119 and 14,392 ASVs were unique in DNA and RNA, respectively. Unique ASVs were generally low abundance (average < 0.000003%) and low prevalence (average < 0.022%) in their respective libraries (Fig. 3). ASVs that were identified in both DNA and RNA were found to be identified at increased though still low abundance (average < 0.00005%) and higher prevalence (average > 0.16%).

**FIG 4:**
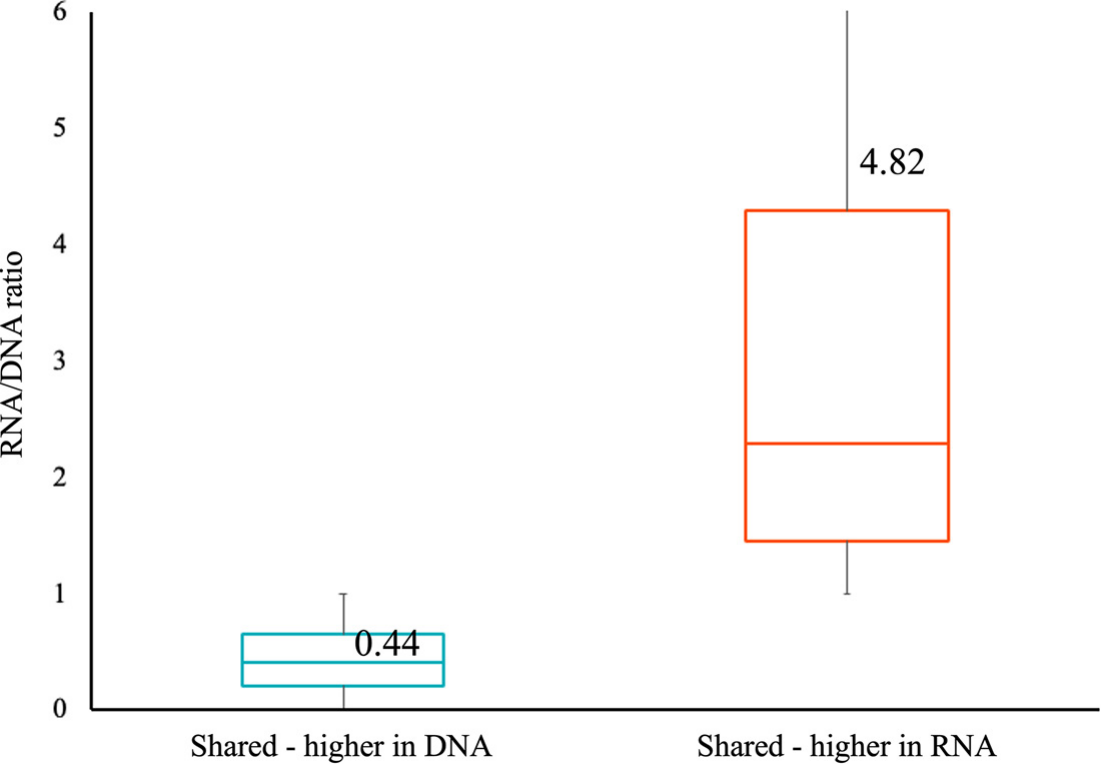
RNA/DNA ratio comparison of the shared ASVs. The ratio of average relative abundance in DNA and RNA microbial communities of ASVs detected in both microbial communities was compared to identify the biased in the DNA- and RNA-based microbial community analysis results. Shared - higher in DNA (blue) and Shared - higher in RNA (red).

ASVs commonly identified between DNA and RNA libraries were further classified based on their enrichment in DNA or RNA, specifically using the ratio of RNA:DNA relative abundances. The RNA:DNA ratio of shared ASVs ranged from 0.0023 to 1,300. The majority of shared ASVs (58%) were more enriched in RNA relative to DNA (Fig. 4). For ASVs enriched in DNA (RNA:DNA ratio < 1), the average RNA:DNA ratio was 0.44; the average RNA:DNA ratio for ASVs enriched in RNA (RNA:DNA ratio > 1) was 4.82. Similar results were found using absolute abundances from total reads observed rather than relative abundances (Fig. S1). Additionally, more variation was observed in shared ASVs which were enriched in RNA relative to those enriched in DNA.

### Phylogenetic composition varied between DNA and RNA microbial communities.

**FIG 5:**
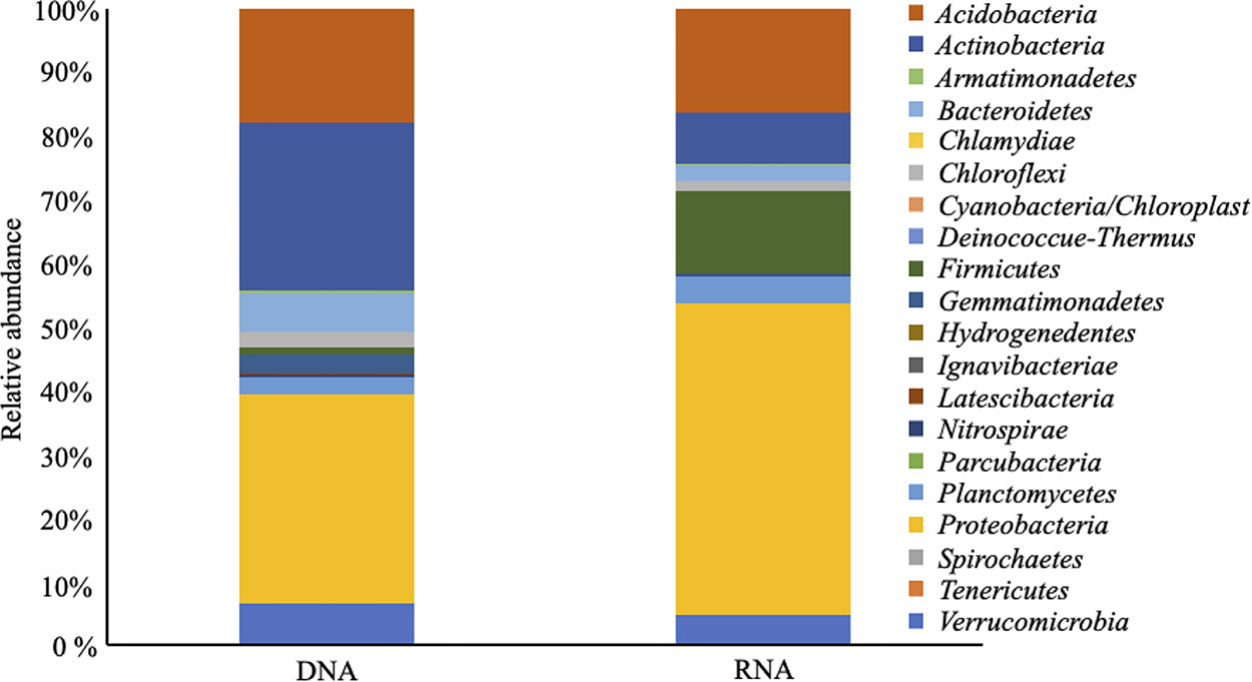
Phylum level differences in DNA and RNA microbial communities. Relative abundances of annotated ASVs are shown, identified to their closest match in the RDP classifier. See Table S2 for a different perspective on the dynamics of numerical relative abundance.

The phylogenetic composition of DNA and RNA microbial communities was compared, with 20 phyla identified in both libraries. Soils were dominated by *Actinobacteria* (26%) and *Proteobacteria* (33%) in DNA and mainly *Proteobacteria* (49%) in RNA (Fig. 5). While DNA and RNA had similar membership at the phylum-level, the relative abundance of every phylum significantly differed (Table S2). Thirteen out of 20 phyla were more enriched in DNA than RNA, and seven phyla were more enriched in the RNA microbial communities.

We evaluated whether the phyla observed to be significantly different between DNA and RNA were comprised of ASVs unique to DNA or RNA or shared between the two methods (Fig. S2). ASV shared by DNA and RNA microbial communities showed more pronounced variations in microbial community structures differences. *Actinobacteria* and *Bacteroidetes* were more enriched in DNA (p_Kruskal-Wallis_ < 0.05), while *Firmicutes* and *Proteobacteria* were more enriched in the RNA microbial community (p_Kruskal-Wallis_ < 0.05). Differentiating ASVs unique in DNA included sequences associated with *Actinobacteria*, *Gemmatimonadetes*, *Latescibacteria*, and *Parcubacteria*. In contrast, sequences associated with *Firmicutes* were unique in RNA.

### DNA and RNA microbial community compositions were variably changed by stand age, N fertilization amount, and time since fertilization.

Previously, the response of the soil microbial community at this site to plant stand age, fertilization history, and time since fertilization was studied based on DNA (43). In this study, subsets of these samples were studied to directly compare DNA and RNA 16S rRNA gene characterization. Based on DNA, community composition responded significantly to the stand age and N fertilization amount. The community response based on RNA was similar, with the notable exception that time since fertilization showed a significant effect only in RNA (Table 1). The effect of stand age and N fertilization amount was generally larger in DNA than RNA, and time since fertilization had a larger effect on the RNA microbial community.

**TABLE 1:** Permutational multivariate analysis of variance (PERMANOVA) for comparing DNA and RNA microbial community dissimilarity

Response variable	Stand age	N fertilization amt	Time since fertilization	Fertilization history
DNA microbial community	R_PERMANOVA_^2^: 0.051	R_PERMANOVA_^2^: 0.015	R_PERMANOVA_^2^: 0.005	R_PERMANOVA_^2^: 0.003
	p_PERMANOVA_: 0.001	p_PERMANOVA_: 0.002	p_PERMANOVA_: n.s	p_PERMANOVA_: n.s
RNA microbial community	R_PERMANOVA_^2^: 0.037	R_PERMANOVA_^2^: 0.009	R_PERMANOVA_^2^: 0.010	R_PERMANOVA_^2^: 0.005
	p_PERMANOVA_: 0.001	p_PERMANOVA_: 0.007	p_PERMANOVA_: 0.008	p_PERMANOVA_: n.s

Next, pairwise comparisons of DNA and RNA microbial communities between stand ages were performed (pairwise PERMANOVA, Table S3). The stand ages of *M.* × *giganteus* included were 2-, 3-, and 4-years-old, and the microbial community of each stand age was significantly different based on both DNA and RNA microbial communities (p_pairwisePERMANOVA_ < 0.05). Similar patterns were observed for the response to N fertilization amount in both libraries, and both DNA and RNA microbial communities were found to have different microbial community compositions for three varying N fertilization amount (p_pairwisePERMANOVA_ < 0.05). Pairwise comparison of DNA and RNA based on sampling day or the time since fertilization resulted in no significant differences observed in DNA, but significant differences between pre-fertilization (10 days before fertilization) and 69 days since fertilization in RNA (Table S4, p_pairwisePERMANOVA_ < 0.05).

**FIG 6:**
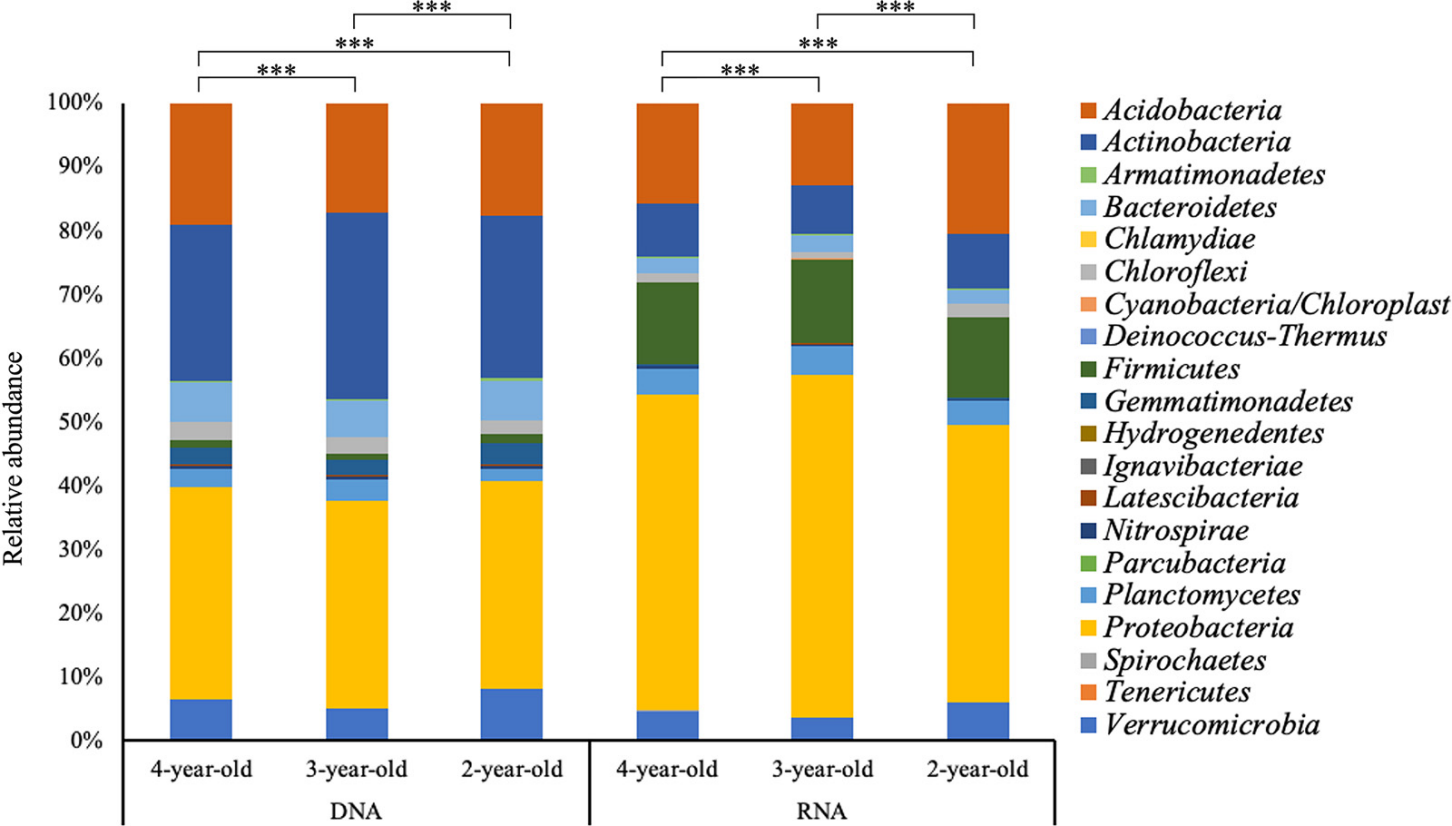
Phylum level differences in DNA and RNA microbial communities according to stand age differences. Relative abundances of annotated ASVs are shown, identified to their closest match in the RDP classifier. “***” denotes significant differences of relative abundance between different stand ages of *M.* × *giganteus* at a *P*-value < 0.05 as assessed by Kruskal-Wallis with *post hoc* Dunn’s test.

Stand age was consistently observed to explain the most variation between experimental factors, regardless of DNA or RNA methods (Table 1). We next evaluated if the specific phyla found to be different between stand ages was consistent between DNA and RNA microbial communities. A total of 20 identical phyla were detected in both sequencing libraries. Every phylum showed significant relative abundance differences between DNA and RNA (p_Kruskal-Wallis_ < 0.05). The dominant phyla differed between DNA and RNA (Fig. 6), with *Acidobacteria* (>17%), *Actinobacteria* (>24%), and *Proteobacteria* (>32%) dominant in DNA, and *Firmicutes* (>12%) and *Proteobacteria* (>43%) in RNA. We subsequently selected these phyla to evaluate genera level differences between DNA and RNA methods.

A total of 569 genera were detected among *Actinobacteria*, *Proteobacteria*, and *Firmicutes*, and 308, 316, and 337 genera in 2-, 3-, and 4-year-old *M.* × *giganteus*, respectively, showed significant differences between the DNA and RNA microbial communities. We selected the genera with greater than 0.1% relative abundance and compared differences between taxonomic profiles in DNA and RNA (Fig. S3). Sequences associated with *Bacillus*, *Clostridium*, *Paenibacillus*, *Sporosarcina* of *Firmicutes* and *Bradyrhizobium*, *Methyloversatilis*, *Nitrosomonas*, *Nitrosospira*, and *Steroidobacter* of *Proteobacteria* were more enriched in RNA than in DNA. On the other hand, *Gaiella* and *Solirubrobacter* of *Actinobacteria* were more enriched in DNA.

Differences in response to fertilization were also observed between DNA and RNA microbial communities. Both DNA- and RNA-based methods identified that soil microbial communities showed different responses to N fertilization amount (Table 1, Table S3), though the phylogenetic profile observed under fertilized conditions differed based on the two methods (Fig. S4). Overall, a greater number of phyla in RNA relative to DNA were significantly affected by differences in N fertilization amount (Table S5). Seven phyla in RNA (*Actinobacteria*, *Firmicutes*, *Gemmatimonadetes*, *Hydrogenedentes*, *Latescibacteria*, *Nitrospirae*, and *Proteobacteria*) showed significant differences between N fertilization amount differences compared with four phyla in DNA (*Acidobacteria*, *Chloroflexi*, *Latescibacteria*, and *Proteobacteria*). *Actinobacteria* (>26%) was more enriched in DNA microbial communities (p_Kruskal-Wallis_ < 0.05), and *Firmicutes* (>12%) and *Proteobacteria* (>47%) were significantly more enriched in RNA microbial communities (p_Kruskal-Wallis_ < 0.05).

Genus-level analysis was performed on the *Actinobacteria*, *Firmicutes*, and *Proteobacteria*, and among the 569 genera detected, 330, 323, and 309 genera showed significant differences between DNA and RNA microbial communities at N fertilization amount of 0, 224, and 448 kg N ha^−1^, respectively (Fig. S5). Sequences associated with *Bacillus*, *Clostridium*, *Paenibacillus*, *Sporosarcina* of *Firmicutes* and *Bradyrhizobium*, *Methyloversatilis*, and *Nitrosomonas* of *Proteobacteria* more enriched in RNA than DNA. On the other hand, *Gaiella* from *Actinobacteria* and *Sphingomonas* of *Proteobacteria* were more abundant in DNA.

### Taxa associated with nitrogen cycle-related bacteria showed a short-term response since fertilization only in RNA microbial communities.

**FIG 7:**
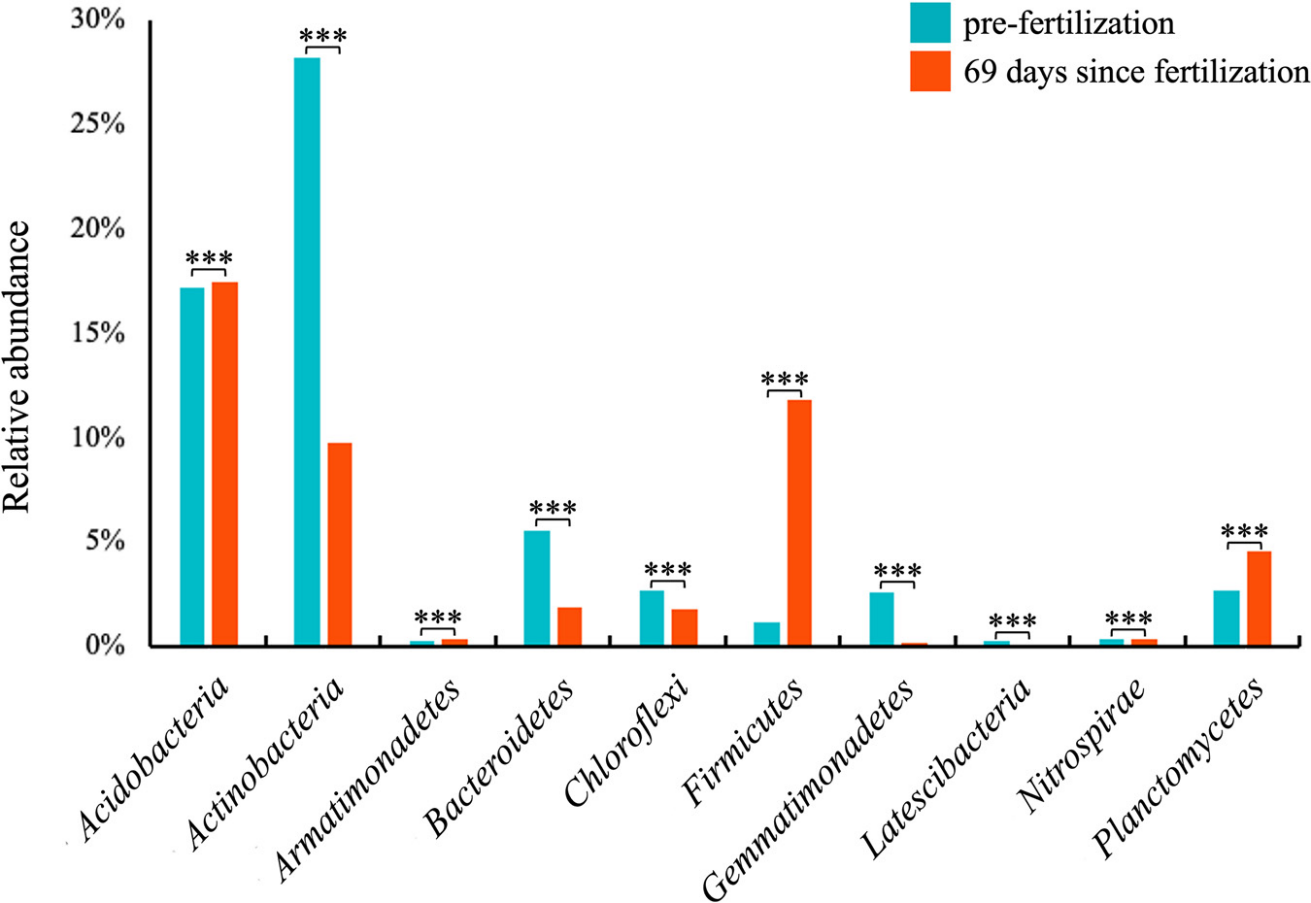
Phylum-level responses to time since fertilization in RNA microbial communities. The average relative abundances of phyla over time since fertilization were summarized. “***” denotes significant differences of relative abundance between pre- and 69 days since fertilization at a *P*-value < 0.05 as assess by Kruskal-Wallis with *post hoc* Dunn’s test.

In comparing pre- and postfertilization soil samples, differences in soil microbial communities were observed only in RNA libraries (Table 1, Table S4). Taxa that were significantly different before and since fertilization were associated with 10 phyla (Fig. 7). Additionally, these differences were only observed 69 days since fertilization, where the relative abundances of *Acidobacteria*, *Armatimonadetes*, *Firmicutes*, and *Planctomycetes* were increased compared with before fertilization, and the relative abundances of *Actinobacteria*, *Bacteroidetes*, *Chloroflexi*, and *Latescibacteria* decreased.

The most enrichment since fertilization was observed in the *Firmicutes*, in which relative abundance was increased 10-fold, and *Planctomycetes* also increased by about 1.7-fold. These phyla are notable because they are known to contain known nitrogen cycling bacteria (53, 54). To better explore the response to fertilization of nitrogen cycling taxa, we obtained taxa that are associated with nitrogen fixation, nitrification, and denitrification from the Fungene database. These taxa included 51 genera associated with *Firmicutes*, *Nitrospirae*, and *Planctomycetes*. We compared the differences of the abundances of these genera between DNA and RNA libraries.

**FIG 8:**
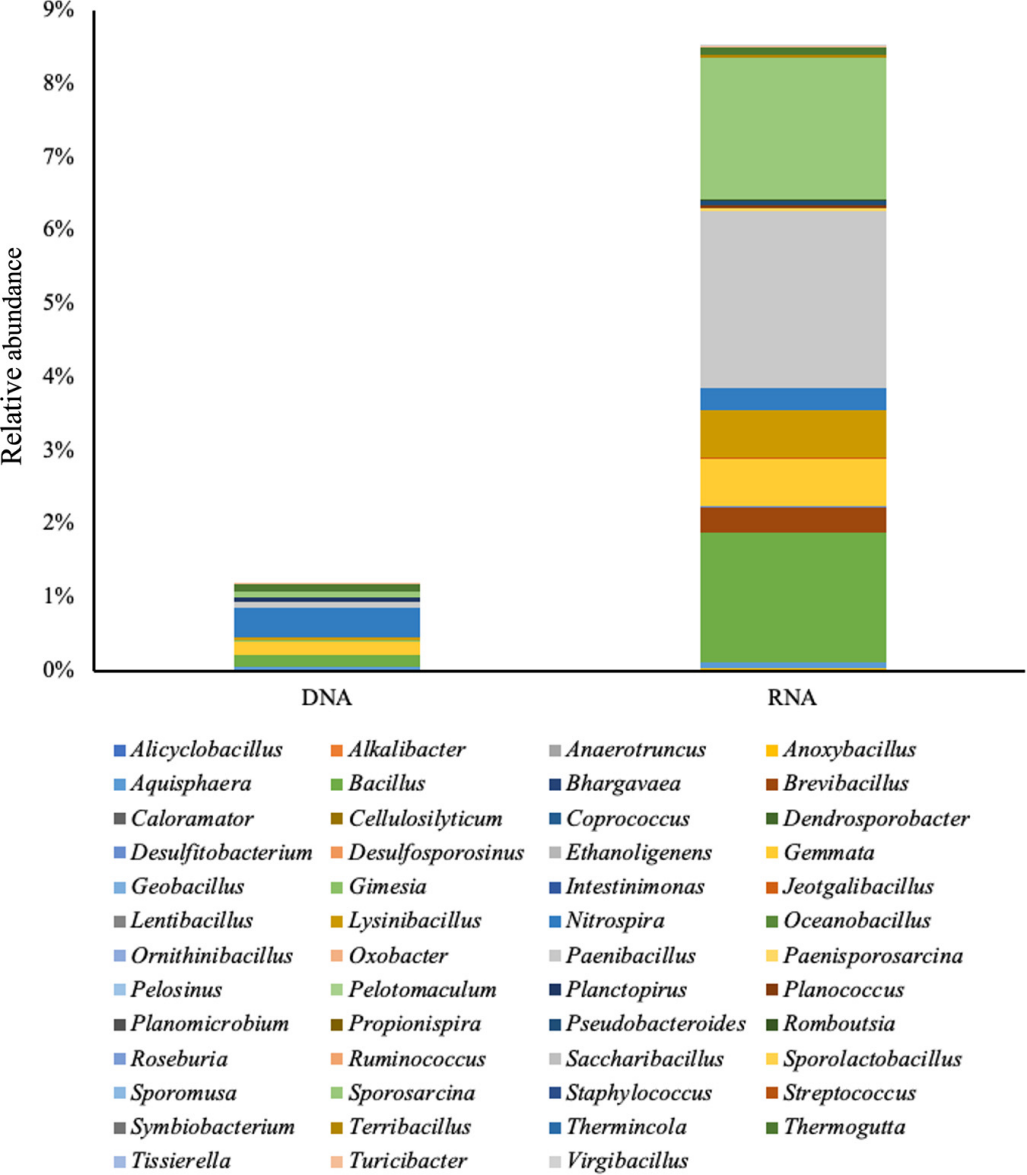
Comparison of nitrogen cycling-related bacteria in the DNA and RNA microbial communities. The average relative abundances of genus associated with nitrogen fixation, nitrification, and denitrification were summarized.

Overall, the total relative abundance of these genera comprised 1.18% and 8.51% in the DNA and RNA microbial communities, respectively (Fig. 8). The large majority of these genera (with the exception of four genera) showed significant differences between DNA and RNA, and among them, *Bacillus*, *Paenibacillus*, and *Sporosarcina* were the most abundant (>20%) in the RNA microbial community.

**FIG 9:**
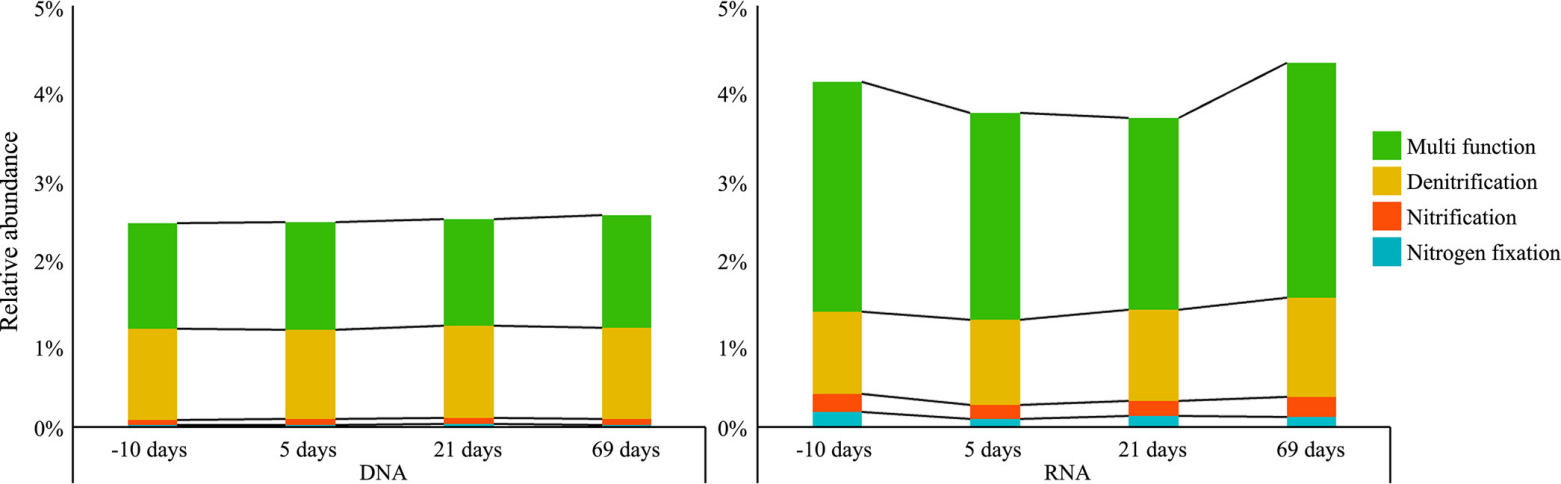
Comparison of nitrogen cycling-related bacteria in the DNA and RNA microbial communities according to time since fertilization. The average relative abundances of bacteria associated with nitrogen fixation, nitrification, and denitrification function are summarized.

The taxa that showed distinct responses in RNA compared with DNA were classified by their known nitrogen cycling functions (excluding taxa with multiple functional annotations). Only taxa associated with denitrification in the RNA microbial communities showed a significant difference (Fig. 9, Table S6) between pre- and postfertilization, consistent with the observation that denitrifying bacteria were consistently enriched since fertilization (55). This response was consistent in *M. × giganteus* from 2-, 3-, and 4-year-old stand ages, where generally RNA showed enrichment of taxa associated nitrogen cycling functions. In all stand ages, DNA was not significantly different pre- and postfertilization within a season. RNA did show seasonal differences, with trends varying depending on stand age (Fig. S6, Table S7).

We further evaluated SSU copy numbers in genes associated with denitrifying phylum that were enriched after fertilization in the RNA microbial communities. Specially, we wanted to understand the potential impact of SSU gene copy numbers on biasing the observed enrichment of these taxa. Overall, we observed differences in the denitrifying phylum enriched in RNA and DNA libraries. In RNA and DNA, the enriched phyla have an average of 2.00 and 1.23 SSU gene copies, respectively (Fig. S7). Among the bacterial membership that were enriched only in RNA microbial communities, *Firmicutes* showed the highest SSU gene copy number of 6.00, followed by *Deinococcus-Thermus*, *Chlamydiae*, and *Actinobacteria*. *Planctomycetes*, *Proteobacteria*, and *Verrucomicrobia* were enriched both DNA and RNA microbial communities, but the average number of SSU gene copies was higher in the taxa of RNA microbial communities.

## DISCUSSION

In direct comparisons of *M.* × *giganteus* soil microbiomes from DNA and RNA extractions, we found that the most significant factor in explaining variation between microbiomes was its sequencing library of origin, even more so than experimental factors of stand age, N fertilization amount, or sampling day (Table S1). DNA and RNA microbiomes also had significantly different alpha diversity, with increased diversity and less variation observed in DNA relative to RNA. These results are consistent with what is known about DNA and RNA. DNA represents the potential genes or membership that may be active and thus is expected to represent more diverse membership with the potential to become metabolically active. RNA, which is actively transcribed, represents growing members, and its higher variability is consistent with its dynamic responses. Previous studies have shown that the RNA microbial community may also have lower alpha diversity because it does not contain the sequences of dormant or dead cells and also has greater variability in response to the environment (49, 56–58).

Overall, most of the membership between DNA and RNA was shared (greater than 90%), suggesting that both methods identify the similar presence of membership. The abundance of these shared membership, however, could be significantly different between DNA and RNA, and most of the shared membership were more enriched in RNA. Based on the assumption that taxa observed in both methods are the most reliable, it is likely that DNA-based methods are underestimating the relative abundance of membership. Further, these differences between DNA and RNA methods contributed to differences in estimated alpha diversity and varying observations of the microbial community response to plant host stand age and fertilization.

In response to both stand age and N fertilization amount, significant differences were observed in both DNA and RNA communities. While the overall pattern and ranking of differences were similar, the magnitude of this change and taxonomic membership driving these differences varied between DNA and RNA approaches. The most significant difference we observed in *M.* × *giganteus* soil microbial communities between the two library methods was in response to nitrogen fertilization. Only RNA microbial communities showed differences pre- and postfertilization and only at day 69. RNA is able to show more rapid changes in response to changes in environmental conditions than DNA (59, 60), and here, we show the ability of RNA to capture a relatively short-term response over the course of one growing season in *M.* × *giganteus*, which is not observed in DNA. These results are consistent with RNA’s short half-life of several minutes to several hours (61) and also justify its usage for measuring short-term seasonal responses in bioenergy soils. Our results showed that it was not until over 2 months that a response different to prefertilization conditions was observed in RNA, providing some insight into the metabolic response of soil microbes to fertilization in these soils. This response was observed in all three stand ages in this study, with youngest 2-year-old stands having the most increase of metabolically active taxa associated with nitrogen-cycling taxa. These results are consistent with previous observations that younger stands are more variable than older stands in their microbial community, potentially due to less maturity of roots and decreased availability of plant litter in younger plants (43).

Among the taxa which were found to be uniquely identified in RNA libraries were members associated with nitrogen-cycling, including members of *Firmicutes*, *Nitrospirae*, and *Planctomycetes* which were enriched with both the presence of fertilizer and increasing nitrogen fertilizer. These results are consistent with previous studies which have shown that *Firmicutes* are enriched when nitrogen fertilizers are used (62–66). In the context of taxa associated with nitrogen cycling, it was confirmed that the RNA-based approach could better detect the denitrification function among the nitrogen cycling functions. This result is consistent with the results of previous studies that the application of nitrogen fertilizers suppressed the activity of nitrogen-fixing bacteria and enhanced denitrifying bacteria (55, 67). These results also emphasize that DNA may underestimate or miss the contribution of nitrogen-cycling taxa, which are highly relevant for nitrogen management in bioenergy systems. In addition to these taxa, we also found that members of dominant soil phyla, *Actinobacteria* and *Proteobacteria* are underestimated using DNA methods alone.

Other factors that may influence differences in DNA and RNA methods include biases from analysis and library preparation. For example, the usage of relative and absolute abundances of taxa may influence comparisons of abundances between DNA and RNA libraries. Relative abundances reflect the proportion of the total potential or metabolically active community, whereas absolute abundances capture the total observations of taxa. In this study, in comparing taxa that were enriched in RNA and DNA, we found similar results with both relative and absolute abundances, indicating that relative or absolute abundances did not significantly bias our observations within this system.

Another factor that should be considered is the variation of copies of SSU among different microbial membership (68). If enriched membership in RNA or DNA libraries carry increased numbers of SSU gene copies, the magnitude of observed differences may be overestimated (69). We evaluated the impact of SSU gene copy numbers on the estimations of abundances of potentially denitrifying taxa enriched in response of fertilization. Our results indicate that phyla enriched in RNA microbial communities are associated with higher SSU gene copy numbers generally, suggesting that they may influence RNA and DNA microbial community comparisons. However, we also observed contrasting phyla in these DNA and RNA libraries, indicating that SSU gene copy number is not the only difference.

Finally, it has previously been observed that RNA preservation methods and chemicals can bias the detection of specific microbial membership. Generally, *Actinobacteria*, *Bacteroidetes*, and *Chloroflexi* have been identified as potentially influenced by RNA storage approaches (70–72). The significant differences of *Actinobacteria* and *Bacteroidetes* between DNA- and RNA-based methods observed in this study and their magnitude are consistent with potential impacts of RNA preservation reported in previous studies. However, we also identify sequences most closely related to *Firmicutes* enriched in RNA (also known nitrogen cycling membership), and there have been no published studies indicating that these are influenced by nucleic acid storage approaches.

In summary, we found that DNA and RNA methods for characterizing the general response of microbial communities varied. While our results support that these variations originate from biological differences, we also acknowledge that they may be influenced by some combination of known (e.g., SSU gene copy number) and unknown biases that would benefit from further research. With relevance to developing sustainable bioenergy crops and understanding the role of microbes in nutrient cycling, RNA appears to better capture the response of taxa known to be involved in nitrogen cycling and is also more sensitive to seasonal shifts in microbiomes. To better link microbial communities to ecosystem processes, we need to move toward characterizing the functional response of microbial communities. Due to costs, the first step in this characterization is often phylogenetic characterization of SSU genes based on DNA. Our results indicate that this method alone may bias against the composition results of the relevant microbial membership.

Notably, the integration of RNA-based methods into an experiment adds significant costs, requiring materials to quickly preserve samples for RNA extraction and typically more time for extraction and library preparation. RNA used for SSU characterization can be a complement to DNA-based studies, as it leverages the advantages and throughput of indicator gene amplification while not being as expensive as metatranscriptomics strategies. Based on our results, we recommend that DNA can be used for the initial and broad characterization of community membership. The use of RNA for SSU characterization could be used to complement DNA characterization when experimental questions have been developed. In the context of our experiment, DNA-based analyses were used to validate that there was a significant response to stand age and fertilization. RNA-based analyses were more helpful in identifying the specific taxa that respond to fertilization. With these specific taxa now identified, future research will be focused on functional characterization, guided by the result of this study (e.g., microbial responses to fertilization responses are most significant 2 months since fertilization). More broadly, in our understanding of microbial ecology, increasing numbers of studies are identifying the environments or gradients for which microbial communities are changing. In future work, it will be necessary to emphasize which taxa or what functions are changing, and our results indicate that RNA-based SSU characterization may be a substantial resource.

## MATERIALS AND METHODS

### Sample description.

Soil samples were collected from the LAMPS site located in Central Iowa, USA (42.013^°^ N, 93.743^°^ W). This staggered-start experiment was planted with *M.* × *giganteus* (clone “Freedom,” AGgrow Tech, High Point, NC, USA) at a density of ∼11 plants m^−2^ in replicated blocks (*n* = 4) in 2015, 2016, and 2017 as described previously (30). The experimental design is a split-plot replicated block with age (planting year) as the main plot and N fertilization rate as the split plot. Soils at the site are deep loams (>1m) formed over glacial till; the dominant soil type (53%) is a Webster clay loam (fine-loamy, mixed, superactive, mesic Typic Endoaquoll). Initially, at this site, soil conditions were considered nitrogen-limiting, with a relatively high C:N ratio (13.2) compared with the average of Northwest Iowa (10.8) (52). Fertilizer was applied as banded urea ammonium nitrate (UAN) in aqueous solution and side-dressed into the soil at 0.1 m depth on May 9, 2018, at rates of 0, 224, and 448 kg ha^−1^ N. Previous recommendations for nitrogen application for miscanthus range from 0 to 120 kg ha^−1^ N (30, 73, 74). The higher fertilization rates used in this study were selected based on the level of nitrogen-limitation in these soils and a parallel study occurring at this site which studied N leaching (52). The rationale for these higher nitrogen fertilization rates were both the level of nitrogen-limitation and parallel studies at this site focused on N leaching from these crops. Soil samples were taken on April 30, May 14, May 30, and July 3, 2018. Soils were collected from within a 10-cm radius of the *M.* × *giganteus* stems using a sampling core (30.5 cm wet sample tube with 1.75 cm diameter, Clements Associates Inc, USA). Soil samples included in this analysis were obtained in triplicate from 60 experimental plots at each time point and analyzed independently. Samples for DNA extraction were stored on dry ice immediately after being taken as described previously (43), and samples for RNA extraction were immediately collected and then frozen in RNAlater (Thermo Fisher Scientific, USA) which offers the advantage of preserving microbial community integrity while preventing RNA degradation (75). All samples were stored in a cooler filled with dry ice during return to the laboratory.

### DNA/RNA extraction and 16S rRNA gene amplicon sequencing.

Plant root tissues in the soil samples were removed to minimize plant nucleic acid contamination. All soil samples were homogenized prior to DNA and RNA extraction and subsampled to 0.25 g. DNA and RNA extraction was performed using the MagAttract PowerMicrobiome DNA/RNA EP Kit (Qiagen, USA) following the standard protocol in this kit and liquid handling in Eppendorf epMotion 5075 (Eppendorf North America). The extracted RNA was transcribed into cDNA according to a standard protocol using iScript cDNA Synthesis Kit (BIO-RAD, USA) for sequencing analysis. The resulting DNA and RNA were analyzed for quantity using an Invitrogen Qubit 4 Fluorometer (Invitrogen, USA). DNA and RNA sample concentrations above 10 ng μL^−1^ were normalized to 10 ng μL^−1^ prior to sequencing. Samples with concentrations lower than 10 ng μL^−1^ were submitted directly for sequencing. The V4 region of the bacterial 16S rRNA gene was amplified with the conserved primers 515F (5′-GTGYCAGCMGCCGCGGTAA-3′) and 806R (5′-GGACTACNVGGGTWTCTAAT-3′) (76, 77). Bacterial amplicon sequencing was performed on Illumina Miseq with Miseq reagent kit V2 (Illumina, USA) at Argonne National Laboratory.

### Amplicon bioinformatics and statistical analysis.

The *DADA2* package (version 1.13.1) in R (version 4.1.0) was used to perform quality control of sequencing libraries and to determine the abundance of ASV. The quality filtering parameters for all sequences were the same as previously described for DNA amplicons (43). The Ribosomal Database Project (RDP) Classifier (version 11.5) was used for taxonomic identification of each observed ASV depending on the sequence similarity to the representatives in the current database. ASVs were removed if no more than 10 total observations were observed in a sample. All statistical analyses were performed in R (version 4.1.0). Two diversity indices, Chao1 and Shannon, were used to compare the alpha diversity of bacteria using the *vegan* package (version 2.5–7). Multivariate homogeneity of group dispersions, calculating the average distance of members to the centroid of the group, was used to analyze the dispersion of each sample using *betadisper* function from the *vegan* package (version 2.5–7). Significant differences in alpha diversity and homogeneity between DNA and RNA microbial communities were evaluated using the Kruskal-Wallis test with Dunn’s *post hoc* test. Permutational multivariate analysis of variance (PERMANOVA) was performed with the *adonis* function of the *vegan* package using the Bray-Curtis dissimilarity matrix (version 2.5–7). PERMANOVA was performed to identify significant differences between centroids of each microbial community, and the R^2^ statistic represents the proportion of the variance for the separation of the microbial community that was explained by experimental and field environmental factors (i.e., origin of the sequencing library, stand age, N fertilization amount, fertilization history, and time since fertilization). PERMANOVA was performed using the “strata” argument for the planted block, which was identified as one of the major factors to structure the microbial composition in the previous study, to better identify the effects of stand age and N fertilization amount, fertilization history, and time since fertilization. This analysis restricted permutations to the data set within each block and was used to quantify variations between and within treatments (43). The comparison between the two groups within the three (stand age, N fertilization amount) or four (time since fertilization) groups was accomplished using pairwise PERMANOVA with the *adonis* function of the *vegan* package (version 2.5–7). The level of significance in the statistical analysis was defined as *P* < 0.05. The rRNA operon copy number database (rrnDB, version 5.7) was used to evaluate SSU gene copy numbers (78). The average SSU gene copy numbers for each phylum were derived according to the phylogenetic classification of microorganisms included in the downloaded database. The results of phylogenetic classification of each ASV in the *M.* × *giganteus* soil microbial communities were subsequently used to calculate the average SSU gene copy number for each phylum in the soil microbial communities.

### Data availability.

The DNA and RNA sequencing data are available at National Center for Biotechnology Information (NCBI) Sequence Read Archive PRJNA601860 and PRJNA745191, respectively.
