# Supplementary material for: Response of Total (DNA) and Metabolically Active (RNA) Microbial Communities in Miscanthus × Giganteus Cultivated Soil to Different Nitrogen Fertilization Rates
Source: Microbiol Spectr. 2022 Feb 16;10(1):e02116-21. doi: 10.1128/spectrum.02116-21 (PMC8849084; doi:10.1128/spectrum.02116-21)
Supplement: SUPPLEMENTAL FILE 1 — Supplemental material. Download SPECTRUM02116-21_Supp_1_seq14.pdf, PDF file, 1.1 MB [file spectrum02116-21_supp_1_seq14.pdf]

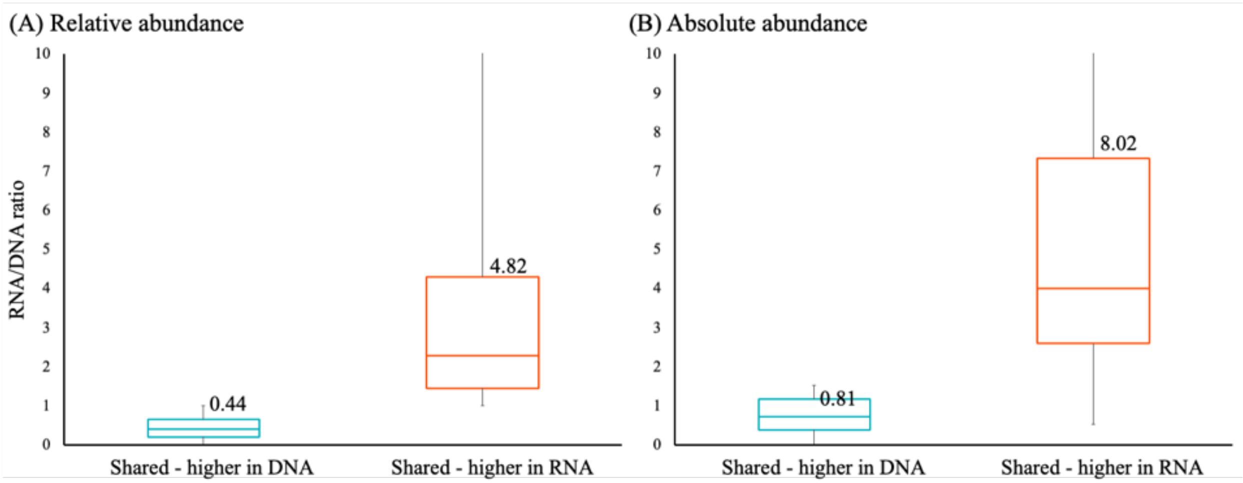

Figure S1. RNA/DNA ratio comparison of the shared ASVs using relative abundance and absolute abundance of ASVs. (A) the ratio of average relative abundance and (B) the ratio of absolute abundance of ASVs in DNA and RNA microbial communities. ASVs detected in both microbial communities showed biased results in the DNA and RNA-based microbial community analysis. Shared - higher in DNA (blue) and Shared - higher in RNA (red).

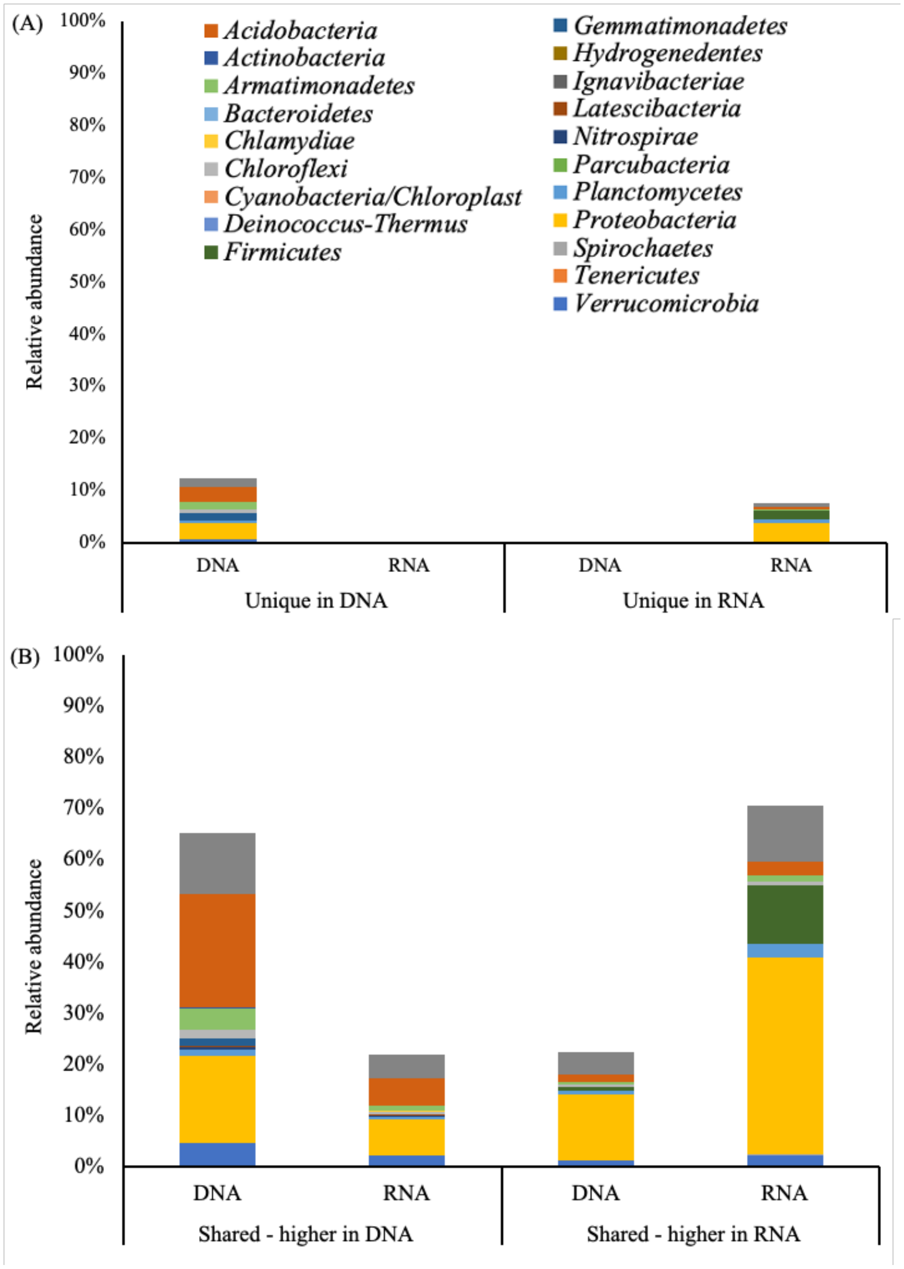

Figure S2. Phylum level differences in DNA and RNA microbial communities based on unique ASVs in DNA or RNA only (A) or shared ASVS and their enrichment in either DNA or RNA (B). Relative abundances of annotated ASVs are shown, identified to their closest match in the RDP classifier.

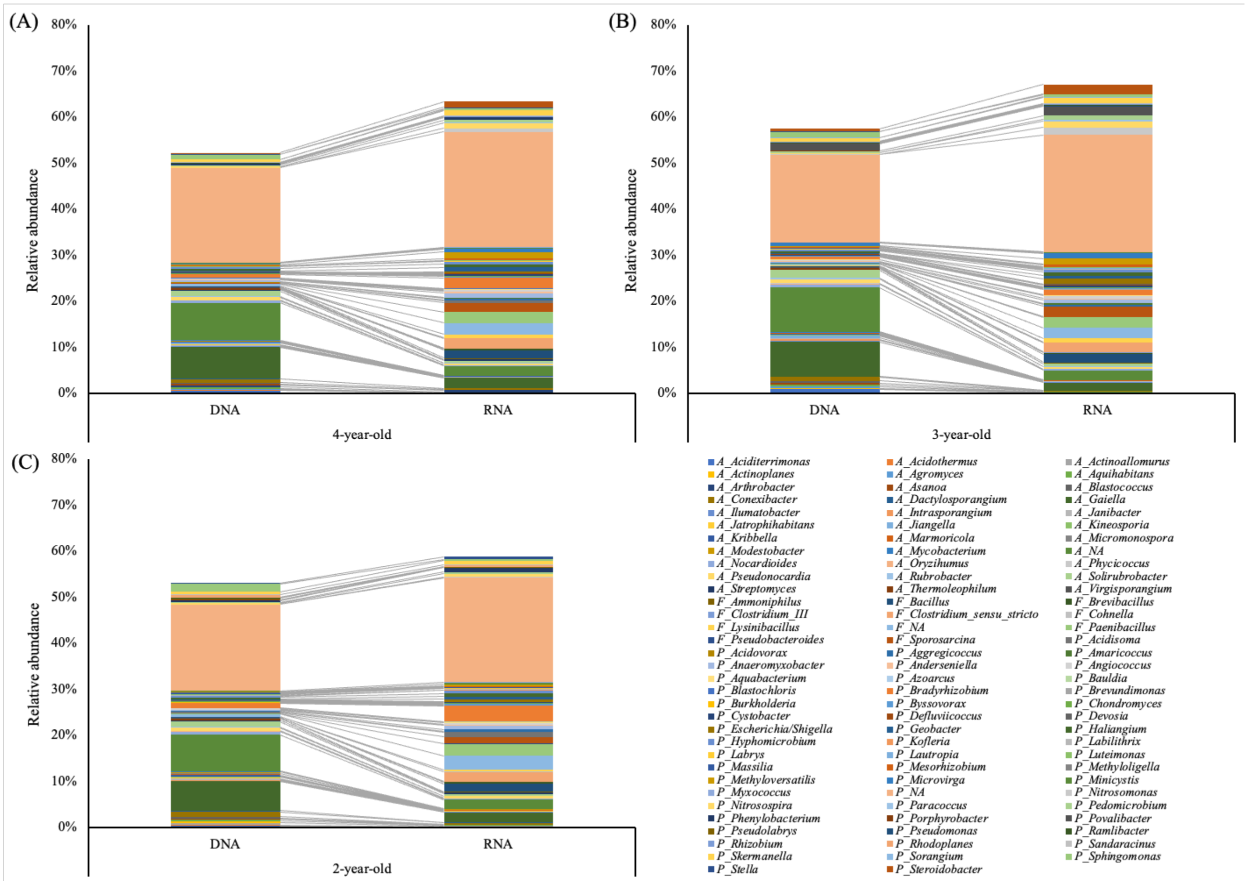

Figure S3. Comparison of dominant genus (> 0.1% relative abundance) in *Actinobacteria*, *Firmicutes*, and *Proteobacteria* between DNA and RNA microbial communities. (A) 4-year-old *M.* x *giganteus*, 73 genera. (B) 3-year-old *M.* x *giganteus*, 77 genera. (C) 2-year-old *M.* x *giganteus*, 74 genera. All genera included this analysis were significantly different between DNA and RNA microbial communities ($p_{\text{Kruskal-Wallis}} < 0.05$).

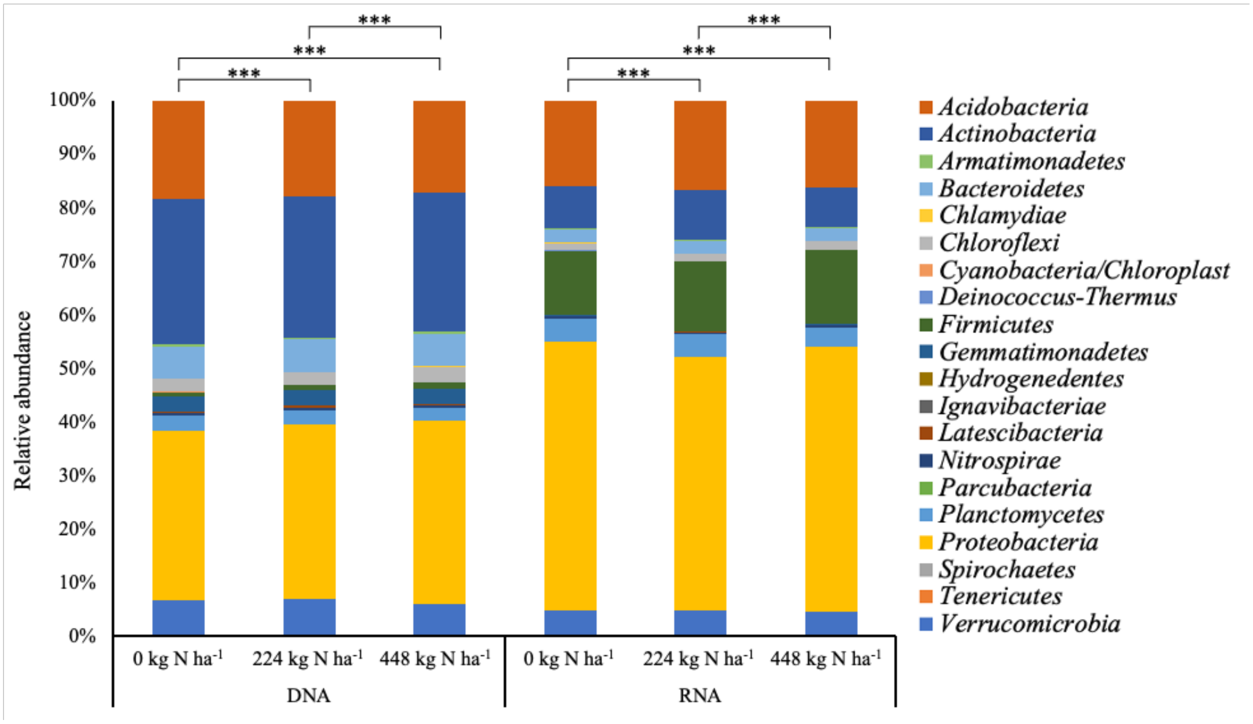

Figure S4. Phylum level differences in DNA and RNA microbial communities according to N fertilization amount differences. Relative abundances of annotated ASVs are shown, identified to their closest match in the RDP classifier. The letters "***" denote significant differences of relative abundance between different stand age of *M.* x *giganteus* at a p-value < 0.05 as assess by Kruskal-Wallis with post hoc Dunn's test.

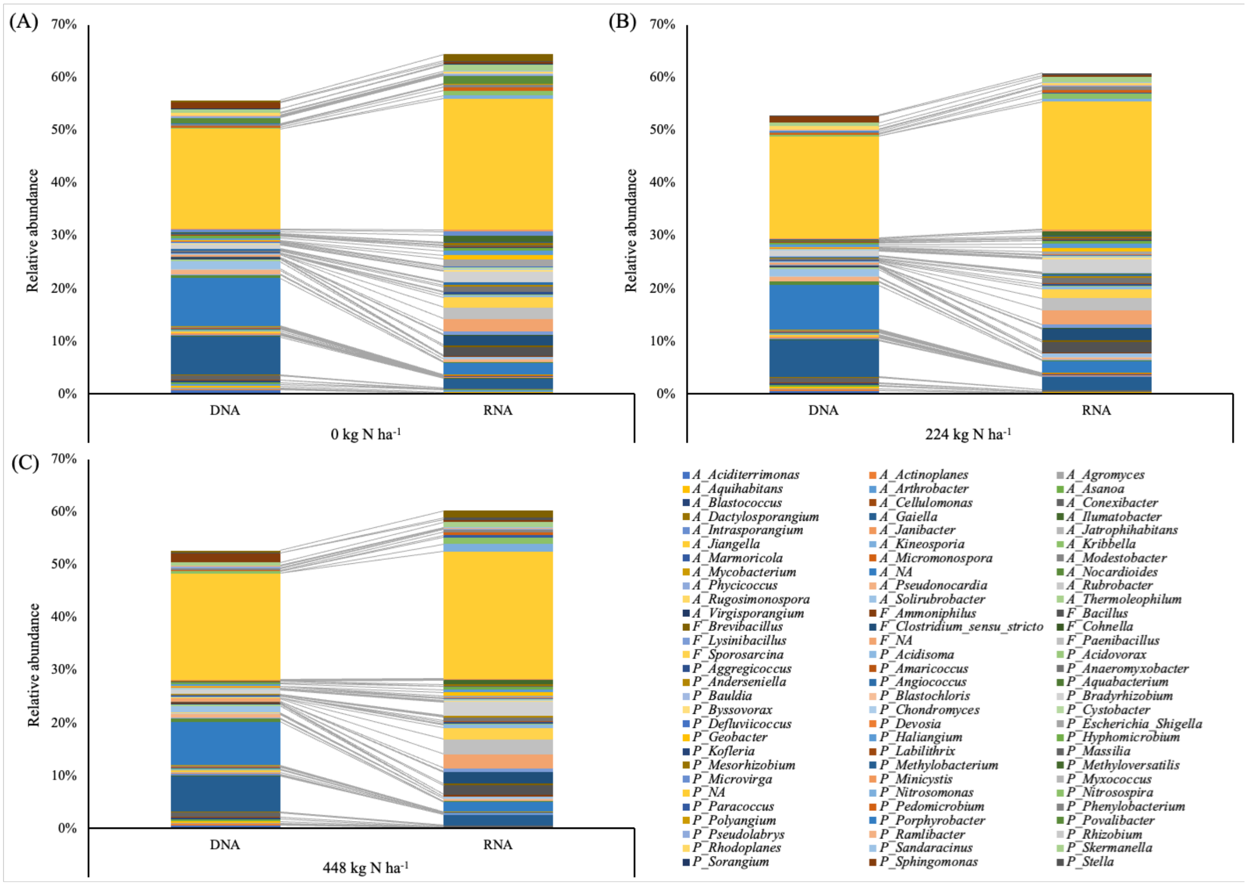

Figure S5. The dynamics of the 88 major genus (> 0.1% relative abundance) in the *Actinobacteria*, *Firmicutes*, and *Proteobacteria* between DNA and RNA microbial communities. (A) 0 kg N ha^{-1} of fertilizer applied *M.* x *giganteus* soil including 79 genera. (B) 224 kg N ha^{-1} of fertilizer applied *M.* x *giganteus* soil including 76 genera. (C) 448 kg N ha^{-1} of fertilizer applied *M.* x *giganteus* soil including 74 genera. All genera included this analysis were significantly different between DNA and RNA microbial communities ($p_{\text{Kruskal-Wallis}} < 0.05$).

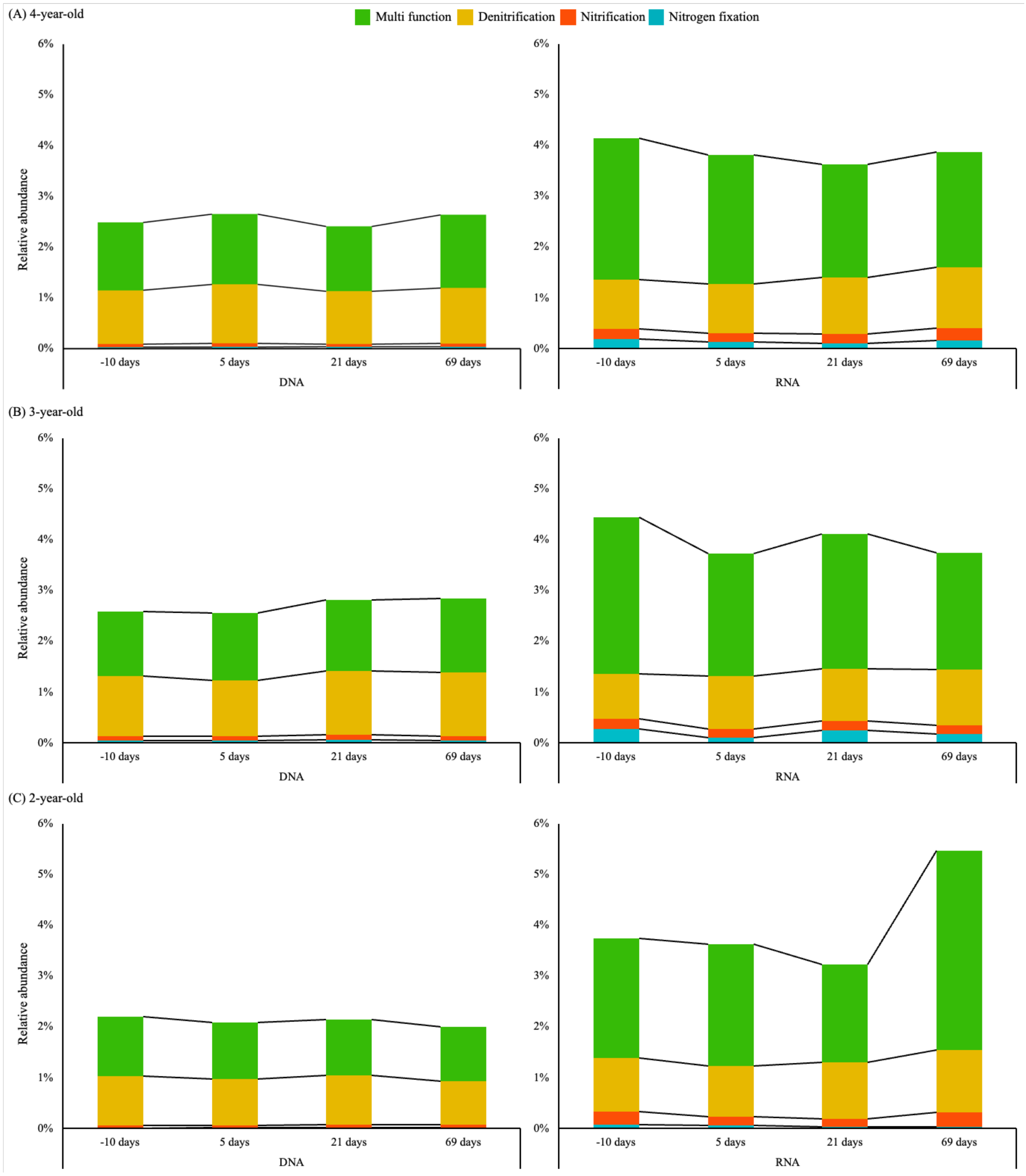

Figure S6. Comparison of nitrogen cycling-related bacteria in the DNA and RNA microbial communities for each stand age according to time since fertilization. (A) 4-year-old *M.* x *giganteus*, (b) 3-year-old *M.* x *giganteus*, and (C) 2-year-old *M.* x *giganteus*. The average relative abundances of bacteria associated with nitrogen fixation, nitrification, and denitrification function were summarized.

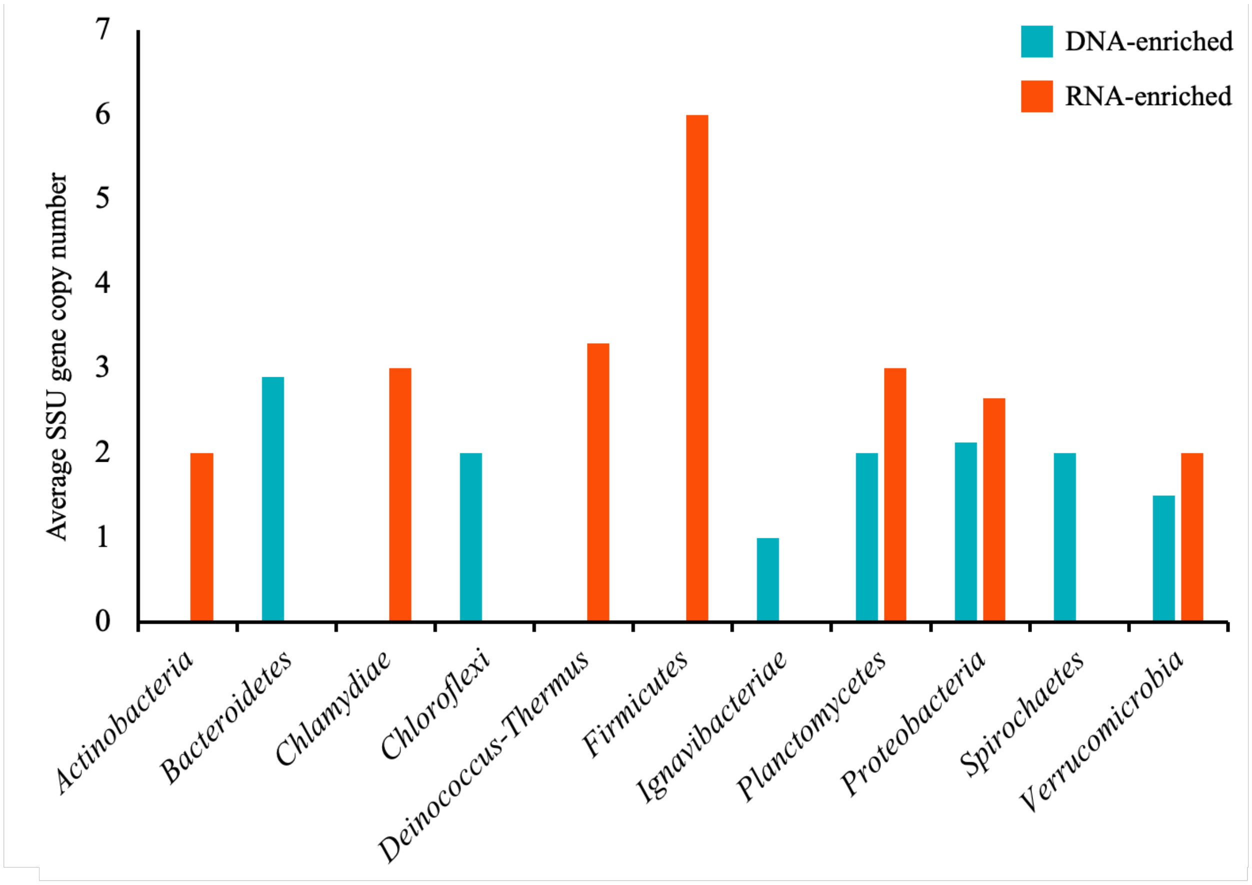

Figure S7. Average SSU gene copy number in bacteria associated with denitrification that were observed to be enriched after fertilization. The taxa that were enriched in the DNA and RNA microbial communities are shown in blue and red, respectively.

Table S1. Comparison of microbial community dissimilarity using permutational multivariate analysis of variance (PERMANOVA) for bacterial communities in *M.* x *giganteus* soil samples.

Response variable	Dissimilarity
Nucleic Acid (DNA and RNA)	$R^2_{PERMANOVA}$: 0.117<br>$p_{PERMANOVA}$: 0.001
Stand ages (2, 3, or 4-year-old)	$R^2_{PERMANOVA}$: 0.030<br>$p_{PERMANOVA}$: 0.001
N fertilization amount (0, 224, and 448 kg N ha^{-1})	$R^2_{PERMANOVA}$: 0.008<br>$p_{PERMANOVA}$: 0.001
Time since fertilization (-10, 5, 21, and 69 days)	$R^2_{PERMANOVA}$: 0.003<br>$p_{PERMANOVA}$: 0.015
Fertilization history (fertilized or unfertilized)	$R^2_{PERMANOVA}$: 0.002<br>$p_{PERMANOVA}$: n.s

Table S2. Kruskal-Wallis with post hoc Dunn's test comparing the average relative abundances of phyla between DNA and RNA microbial communities.

Phylum	Average relative abundance		p-value
	DNA	RNA	
Acidobacteria	17.88%	16.29%	6.54E-09
Actinobacteria	26.46%	8.18%	2.22E-87
Armatimonadetes	0.31%	0.23%	1.92E-17
Bacteroidetes	6.02%	2.34%	1.13E-77
Chlamydiae	0.05%	0.10%	1.60E-93
Chloroflexi	2.58%	1.47%	3.04E-90
Cyanobacteria/Chloroplast	0.02%	0.03%	7.67E-93
Deinococcus-Thermus	0.00%	0.02%	4.75E-07
Firmicutes	1.05%	12.90%	1.13E-92
Gemmatimonadetes	2.81%	0.14%	1.33E-87
Hydrogenedentes	0.00%	0.00%	7.75E-83
Ignavibacteriae	0.01%	0.01%	2.61E-11
Latescibacteria	0.28%	0.10%	4.30E-83
Nitrospirae	0.45%	0.33%	1.69E-74
Parcubacteria	0.05%	0.00%	7.05E-68
Planctomycetes	2.64%	4.06%	4.49E-98
Proteobacteria	32.76%	48.97%	3.08E-90
Spirochaetes	0.01%	0.01%	1.50E-94
Tenericutes	0.00%	0.00%	1.93E-33
Verrucomicrobia	6.63%	4.81%	3.18E-15

Table S3. Pairwise permutational multivariate analysis of variance (PERMANOVA) for comparing the effect of stand age and fertilization on the DNA and RNA microbial community dissimilarity.

Response variable	Stand age		N fertilization amount (kg N ha^{-1})	
DNA microbial community	4-year-old vs 3-year-old	$R^2_{pairwisePERMANOVA} = 0.063$ $p_{pairwisePERMANOVA} = 0.001$	0 vs 224	$R^2_{pairwisePERMANOVA} = 0.014$ $p_{pairwisePERMANOVA} = 0.023$
	4-year-old vs 2-year-old	$R^2_{pairwisePERMANOVA} = 0.073$ $p_{pairwisePERMANOVA} = 0.001$	0 vs 448	$R^2_{pairwisePERMANOVA} = 0.021$ $p_{pairwisePERMANOVA} = 0.018$
	3-year-old vs 2-year-old	$R^2_{pairwisePERMANOVA} = 0.210$ $p_{pairwisePERMANOVA} = 0.001$	224 vs 448	$R^2_{pairwisePERMANOVA} = 0.015$ $p_{pairwisePERMANOVA} = 0.023$
RNA microbial community	4-year-old vs 3-year-old	$R^2_{pairwisePERMANOVA} = 0.039$ $p_{pairwisePERMANOVA} = 0.001$	0 vs 224	$R^2_{pairwisePERMANOVA} = 0.011$ $p_{pairwisePERMANOVA} = 0.031$
	4-year-old vs 2-year-old	$R^2_{pairwisePERMANOVA} = 0.057$ $p_{pairwisePERMANOVA} = 0.001$	0 vs 448	$R^2_{pairwisePERMANOVA} = 0.013$ $p_{pairwisePERMANOVA} = 0.029$
	3-year-old vs 2-year-old	$R^2_{pairwisePERMANOVA} = 0.152$ $p_{pairwisePERMANOVA} = 0.001$	224 vs 448	$R^2_{pairwisePERMANOVA} = 0.012$ $p_{pairwisePERMANOVA} = 0.029$

Table S4. Pairwise permutational multivariate analysis of variance (PERMANOVA) for comparing the effect of time since fertilization on the DNA and RNA microbial community dissimilarity.

Response variable	Time since fertilization	
DNA microbial community	-10 vs 5	n.s
	-10 vs 21	n.s
	-10 vs 69	n.s
	5 vs 21	n.s
	5 vs 69	n.s
	21 vs 69	n.s
RNA microbial community	-10 vs 5	n.s
	-10 vs 21	n.s
	-10 vs 69	$R^2_{pairwisePERMANOVA} = 0.018$, $p_{pairwisePERMANOVA} = 0.018$
	5 vs 21	n.s
	5 vs 69	n.s
	21 vs 69	n.s

Table S5. Kruskal-Wallis with post hoc Dunn's test comparing the average relative abundances of phyla between DNA and RNA microbial communities by different N fertilization. Denote N0, N224, and N448 are N fertilization of 0 kg N ha^{-1}, 224 kg N ha^{-1}, and 448 kg N ha^{-1}, respectively.

	Phylum	Difference in average relative abundance		p-value
DNA microbial community	*Acidobacteria*	18.44%	17.23%	3.62E-02 between N0 and N448
	Chloroflexi	2.30%	2.90%	1.24E-02 between N224 and N448
	Latescibacteria	0.32%	0.30%	1.79E-04 between N0 and N448
		0.30%	0.21%	1.30E-03 between N224 and N448
	Proteobacteria	31.54%	34.10%	3.17E-04 between N0 and N448
RNA microbial community	*Actinobacteria*	9.18%	7.39%	1.33E-03 between N224 and N448
	Firmicutes	12.91%	13.78%	2.55E-03 between N224 and N448
	Gemmatimonadetes	0.12%	0.13%	4.52E-02 between N0 and N448
	Hydrogenedentes	0.0054%	0.0047%	2.47E-02 between N0 and N224
		0.0047%	0.0035%	2.62E-02 between N224 and N448
	Latescibacteria	0.10%	0.08%	3.87E-03 between N224 and N448
	Nitropirae	0.30%	0.31%	1.59E-04 between N0 and N448
	Proteobacteria	50.08%	49.46%	3.27E-05 between N0 and N448

Table S6. Kruskal-Wallis with post hoc Dunn's test comparing the average relative abundances of nitrogen cycling functions in RNA microbial communities by time since fertilization.

	Function	Days		Relative abundance		p-value
RNA microbial community	Denitrification	-10 days	69 days	0.85%	1.15%	1.52E-08
		5 days	69 days	0.95%	1.15%	7.72E-03
		21 days	69 days	0.94%	1.15%	2.72E-03

Table S7. Kruskal-Wallis with post hoc Dunn's test comparing the average relative abundances of nitrogen cycling functions in RNA microbial communities for each stand age by time since fertilization.

Stand age	Function	Days		Relative abundance		p-value
4-year-old	Denitrification	-10 days	69 days	0.87%	1.16%	1.17E-02
		21 days	69 days	0.88%	1.16%	3.80E-02
3-year-old		-10 days	5 days	0.81%	1.09%	1.52E-03
		-10 days	21 days	0.81%	1.03%	1.15E-02
		-10 days	69 days	0.81%	1.07%	3.93E-03
2-year-old		-10 days	69 days	0.87%	1.22%	9.17E-04
		5 days	69 days	0.90%	1.22%	7.07E-03
		21 days	69 days	0.88%	1.22%	5.34E-03
